# A broad pH range and processive chitinase from a metagenome library

**DOI:** 10.1590/1414-431X20165658

**Published:** 2017-01-05

**Authors:** S.S. Thimoteo, A. Glogauer, H. Faoro, E.M. de Souza, L.F. Huergo, B.M. Moerschbacher, F.O. Pedrosa

**Affiliations:** 1 Departmento de Bioquímica e Biologia Molecular, Universidade Federal do Paraná, Curitiba, PR, Brasil; 2 Agência de Inovação, Instituto de Tecnologia do Paraná - Tecpar, Curitiba, PR, Brasil; 3 Instituto Carlos Chagas, Fiocruz, Curitiba, PR, Brasil; 4 Institute for Biology and Biotechnology of Plants, WWU Münster University, Münster, Germany

**Keywords:** Chitinase, Metagenomic, Aeromonas, Kinetics

## Abstract

Chitinases are hydrolases that degrade chitin, a polymer of N-acetylglucosamine linked β(1-4) present in the exoskeleton of crustaceans, insects, nematodes and fungal cell walls. A metagenome fosmid library from a wastewater-contaminated soil was functionally screened for chitinase activity leading to the isolation and identification of a chitinase gene named *metachi18A*. The *metachi18A* gene was subcloned and overexpressed in *Escherichia coli* BL21 and the MetaChi18A chitinase was purified by affinity chromatography as a 6xHis-tagged fusion protein. The MetaChi18A enzyme is a 92-kDa protein with a conserved active site domain of glycosyl hydrolases family 18. It hydrolyses colloidal chitin with an optimum pH of 5 and temperature of 50°C. Moreover, the enzyme retained at least 80% of its activity in the pH range from 4 to 9 and 98% at 600 mM NaCl. Thin layer chromatography analyses identified chitobiose as the main product of MetaChi18A on chitin polymers as substrate. Kinetic analysis showed inhibition of MetaChi18A activity at high concentrations of colloidal chitin and 4-methylumbelliferyl N,N′-diacetylchitobiose and sigmoid kinetics at low concentrations of colloidal chitin, indicating a possible conformational change to lead the chitin chain from the chitin-binding to the catalytic domain. The observed stability and activity of MetaChi18A over a wide range of conditions suggest that this chitinase, now characterized, may be suitable for application in the industrial processing of chitin.

## Introduction

Chitin provides structural support and protection for numerous organisms. It is a common constituent of insect exoskeletons, crustacean shells, some algae cell walls and many agronomically important pathogens and pests including fungi and nematodes, but it is absent in higher plants and animals. Chitin is the second most abundant biopolymer on Earth, exceeded only by cellulose (1). It is composed of linear chains of β-1,4-linked N-acetylglucosamine (GlcNAc) residues that can assemble in a crystalline structure by many intramolecular hydrogen bonds turning insoluble, similar to cellulose.

Annually, about 6 to 8 million tonnes of crustacean shells are wasted worldwide, from which 15–40% corresponds to chitin (2). The enormous amount of chitin and chitosan continuously generated in nature and from human consumption requires disposal and recycling on a formidable scale. The complete enzymatic hydrolysis of chitin to free GlcNAc involves lytic polysaccharide monooxygenases (3) and glycoside hydrolases [EC.3.2.1.14]; the latter are classified by amino acid sequence homology into families 18, 19 and 20. The chitinases of glycosyl hydrolases in family 20 (GH-20) are β-(1,4)-N-acetyl-glucosaminidases that release GlcNAc monomers (4).

Families 18 (GH-18) and 19 (GH-19) chitinases differ in their structure and catalytic mechanism. Family 19 chitinases have predominance of α-helix fold and an inverting, single-displacement catalytic mechanism, whereas family 18 chitinases have a (β/α)_8_-barrel fold as the catalytic domain and may have additional carbohydrate binding modules (CBM) or fibronectin type III-like domains (Fn3). Their catalytic mechanism is a substrate-assisted double displacement with retention of substrate conformation (5).

The catalytic domain of family 18 chitinases has conserved sequences SXGG for binding and DXDXE for hydrolysis, with the glutamate residue participating as proton donor at the -1 catalytic subsite. The catalytic domains of family 18 can be divided into three subfamilies, A, B and C, with family A containing an additional (α+β) fold inserted between the seventh and eighth β-strand. This additional domain creates a deeper catalytic cleft facilitating longer chitin chain hydrolyses (6).

Accessibility to the chitin chain is facilitated by the chitin-binding domains, such as the CBM and Fn3 domain. Substrate binding may occur at the reducing or non-reducing ends of the chitin chain for exochitinases, or randomly along the chain for endochitinases. Both types of chitinases can display a processive mode of action, releasing a series of oligomers (mostly dimers) before dissociating from the substrate. Usually, processive chitinases have a deep catalytic cleft and a path of aromatic amino acid residues from the chitin-binding domain to the catalytic domain. This path helps in the correct positioning of substrate on the catalytic subsites, and the hydrophobic interactions give a strong but flexible binding to guide the chitin chain into the active site (1,7).

Traditionally, the enzymes used by the industry are derived from cultivable microorganisms. This has certainly limited the discovery of novel enzymes with potential for industrial applications (8), since more than 99% of microorganisms present in the environment cannot be cultivated using available methods (9). To overcome this limitation, a strategy that involves the direct cloning of the total microbial genomes (metagenome) from the environment into a cultivable host such as *Escherichia coli* was developed (10). The metagenomic strategy has been successfully employed to isolate and identify enzymes through functional screening, PCR approaches or DNA sequencing followed by homology searches.

The presence and diversity of chitinases have been investigated in some metagenome libraries from diverse environments such as soil (11), aquatic habitats (12) and extreme habitats (13) using PCR or sequencing approaches. However, only a few works went further on the characterization of found chitinases.

In this study, a metagenomic fosmid library was functionally screened for chitinase activity, and a chitinase coding gene, named *metachi18A*, was identified and cloned into an expression vector. The purified recombinant chitinase was active on a range of chitin polymers over a wide range of physico-chemical conditions, suggesting that this novel chitinase may be suitable for biotechnological applications.

## Material and Methods

### Bacterial strains and plasmids

*Escherichia coli* EPI300TM-T1R and pCC2FOS fosmid vector (CopyControl Fosmid Library Production Kit, Epicentre Biotechnologies, USA) were used in the metagenomic library. *E. coli* DH10B and the vectors pUC18 and pCR2.1 (Invitrogen Life Technologies, USA) were used for subcloning steps. *E. coli* BL21(DE3) and vector pET-28a(+) (Novagen, USA) were used as the recombinant protein expression system.

### Chemicals and enzymes

FideliTaq PCR Master Mix (USB, USA) was used for DNA amplification. T4 DNA ligase, T4 DNA polymerase, Klenow fragment, T4 polynucleotide kinase, shrimp alkaline phosphatase (SAP), restriction enzymes and the protein molecular mass marker were purchased from Fermentas (USA). The HiTrap Chelating HP column was purchased from GE Healthcare (Uppsala, Sweden). Chitin from crab shells and Fluorimetric Chitinase Assay Kit were purchased from Sigma-Aldrich (USA). Polyglucosamine (DA 0%) used for the preparation of partially acetylated chitosans as well as α- and β-chitin were kindly provided by Mahtani Chitosan (India). All other chemicals used for chitinase analysis were of analytical grade.

### Colloidal chitin preparation

Colloidal chitin was prepared according to the protocol described by Hsu and Lockwood (14) with some modifications. Chitin from crab shells was ground in a ball mill and selected for 40 mesh. Twenty grams of chitin powder were gently stirred with 200 mL of 85% phosphoric acid and allowed to rest for 24 h at 4°C. Colloidal chitin was washed with tap water four times. The pH was adjusted to 7 for the last wash, and the material obtained was stored at 4°C.

### Metagenomic library and screening for chitinolytic activity

The metagenomic library constructed by Glogauer et al. (15) in fosmid pCC2FOS (around 500,000 clones) was manually screened for chitinase activity using Luria-Bertani Agar (LA) with 2% colloidal chitin. The metagenomic library clones were collected into a single pool, which was serial diluted and plated on LA-chitin, comprising 50,000 colonies distributed in 50 Petri dishes (Ø=150 mm). After incubation at 37°C for 7 days and at room temperature for another 10 days, clones with hydrolysis halos were selected. Fosmids were purified by the alkaline lysis method and the restriction cut patterns of *Eco*RI and *Bam*HI were analyzed. Single-pattern fosmids were retransformed into *E. coli* EPI300 and their chitinolytic activity on LA-chitin plates was reevaluated.

### Subcloning and identification of the chitinase gene

The FosChit DNA was isolated and mechanically fragmented. The fragments were separated by agarose gel electrophoresis and the fragments of 3 and 5 Kb were purified and cloned in pUC18 or pCR2.1 plasmid. This sublibrary of the FosChit was screened for chitinolytic activity on modified LA-chitin plates. Both ends of the inserts of active subclones were sequenced on an ABI 3500xL Genetic Analyzer (Applied Biosystems, USA) automated sequencer using Big Dye Terminator Kit and pUC M13 primers (Applied Biosystems). The plasmids of active clones were also submitted to a random insertion of EZ-Tn5<KAN-2> obtained by *in vitro* transposon insertion reaction with the EZTn5<KAN-2> Insertion Kit (Epicentre). Both ends of the insertion regions of 96 subclones were also sequenced using the transposon forward and reverse primers provided with the insertion kit. Sequence assembly and editing were performed with the Phred-Phrap-Consed software (16). The amino acid sequences were compared with the non-redundant sequence database deposited at NCBI using BLAST.

### Chitinase sequence analyses

Predictions of signal peptide sequences were performed using SignalP 3.0 (17). The ProtParam tool was used to calculate the theoretical parameters of the protein (18). A multiple sequence alignment was performed using ClustalW algorithm (19). Predicted domains were analyzed by BLASTp (NCBI).

Structures of proteins with sequences similar to MetaChi18A were acquired on Protein Data Bank (PDB) (20). The sequence and secondary structure of two chitinases were compared with sequence MetaChi18A. A multiple sequence alignment was performed using ClustalW algorithm (19) and the assignments of secondary structures were performed with the DSSP program (21) using PDB entries. The visualization and edition of sequences and secondary structures were performed on ALINE software (22).

### Cloning of gene *metachi18A*

A pair of primers was designed based on the assembled sequence of the active subclones to amplify gene *metachi18A* with restriction sites at both ends and to generate a chitinase with a C-terminal His-tag when expressed in the pET28 vector. Forward primer MetaChi18AFor (5′ TACAACCATGGCAAGTCCAAAACCT 3′) and reverse primer MetaChi18ARev (5′ AGCGGAAGCTTGTACTTGCAGCTG 3′) were used in a PCR reaction (FideliTaq PCR Master Mix, USB, USA) for the amplification of a 2637 bp fragment. The amplified gene was first cloned into the pCR 2.1 vector (TA Cloning Kit, Invitrogen, USA) according to the manufacturer’s recommendations, and recombinant plasmids were transformed into *E. coli* DH10B competent cells by electroporation. The inserts were sequenced with M13 forward and reverse primers and with designed primers, internal to the chitinase gene, to confirm the absence of mutations in MetaChi18A. Recombinant plasmid was then digested with *Nco*I (cut at the MetaChi18A translation start codon) and *Hind*III. The insert was ligated into vector pET28a(+), which had been previously digested with the same restriction enzymes and dephosphorylated by SAP, yielding plasmid pET28a-MetaChi18A – the insert of which was confirmed by end-sequencing using T7 promoter and T7 terminator primers. Plasmid pET28a-MetaChi18A was then transformed into *E. coli* BL21(DE3) cells to express the recombinant C-terminal (His)6-tagged MetaChi18A chitinase.

### Overexpression and purification of recombinant MetaChi18A chitinase

*E. coli* BL21(DE3) cells carrying the pET28a-MetaChi18A plasmid were grown in 200 mL of LB medium at 37°C until an OD600 of 0.5, and induced by the addition of isopropyl-β-D thiogalactopyranoside (IPTG) to a final concentration of 0.3 mM. The induced culture was incubated for a further 3 h at 30°C before the harvesting of the cells by centrifugation (10,000 *g* for 5 min) at 4°C. The cell pellet was suspended in 20 mL of lysis buffer (20 mM Tris-HCl, pH 8.0, 150 mM NaCl) and disrupted by ultrasonication in an ice bath (10 cycles of 40 s pulses, 90 W, with 20 s intervals), using a Sonicator® XL 2020 (Heat Systems-Ultrasonics Inc., USA). The crude extract was then centrifuged at 20,000 *g* for 30 min at 4°C to pellet the cell debris. The supernatant containing the His-tagged protein was loaded onto a HiTrap Chelating HP 5 mL column (GE Healthcare, USA), previously loaded with NiCl_2_ 100 mM and equilibrated with lysis buffer, using an ÄKTA basic chromatography system (GE Healthcare). The column was washed with 5 volumes of the lysis buffer. The His-tagged protein was eluted with an increasing gradient of imidazole up to 500 mM in elution buffer. The elution of protein was monitored at 280 nm and protein fractions were analyzed by SDS-PAGE, pooled, dialyzed (20 mM Tris-HCl, pH 8.0, 150 mM NaCl, 50% (v/v) glycerol) and stored at −24°C until use.

### Protein content determination, electrophoresis and zymogram analyses

Protein content was determined using Bradford Protein Assay Reagent, following the manufacturer’s protocol (Bio-Rad, Brazil) with bovine serum albumin as the standard. Electrophoresis of protein samples was done with 10% (w/v) SDS-PAGE and the gel was stained with Coomassie brilliant blue R-250 and destained with methanol/acetic-acid/water (5/1/4 v/v/v). Densitometry analysis of the stained SDS-PAGE gel was performed with LabWorks Image Acquisition and Analysis Software 4.0 (UVP BioImaging Systems, USA).

For zymogram analyses, the purified protein was mixed with a loading buffer without reducing agent, heated at 90°C for 5 min, and applied onto two wells of the gel. After separation, the gel was sliced in two. One part was submerged in Coomassie brilliant blue R-250 for protein bands visualization and the other part was renatured. The protein was renatured by removing SDS with two washes of 20 mM sodium acetate buffer, pH 5.0, and incubation in 50 mL of 20 mM sodium acetate buffer with 2.5% Triton X-100 (v/v) for 3 h, with gentle agitation. The gel was then rinsed in 50 mM sodium acetate buffer, pH 5.0, and incubated in the same buffer for 20 min. A substrate layer was prepared with 1% agar supplemented with 1% colloidal chitin in 20 mM sodium acetate buffer, pH 5.0, (23). Enzyme activity was detected by overlaying the substrate gel onto the polyacrylamide gel, in a Petri plate, followed by incubation at 37°C overnight. Bands exhibiting chitinolytic activity were visualized as clearing zones on substrate gel.

### MALDI-TOF/MS analysis of purified MetaChi18A

Matrix-assisted laser desorption/ionization (MALDI) time-of-flight (TOF) mass spectra (MS) were acquired on a MALDI-TOF/TOF Autoflex II spectrometer (Bruker Daltonics, Germany) in the reflector positive ion mode with an acceleration voltage of 20 kV, a delay time of 150 ns and an acquisition mass range of 800 to 3200 Da. Spots were manually excised from SDS-PAGE and digested in-gel with sequencing grade modified trypsin (Promega, USA) as described elsewhere (24). The sample was desalted using a ZipTipC18 pipette tip (Millipore Corporation, USA) and eluted directly onto the MALDI target plate using MALDI matrix (saturated solution of a-cyano-4-hydroxycinnamic acid in 50% (v/v) acetonitrile and 0.1% TFA). Mass profiles were identified by comparing the peptide masses obtained with *in silico* digestion of the His-tagged protein sequence using PeptideCutter and MS- Digest tools (18).

### Chitinase activity assay using 4-MUF-chitooligosaccharides

Chitinase activity was tested using a fluorimetric assay following the manufacturer’s protocol (Sigma CS1030). Forty nanograms of enzyme were added to 90 µL of assay buffer containing as a substrate 0.2 mg/mL 4-MUF-GlcNAc, 4-MUF-(GlcNAc)_2_ or 4-MUF-(GlcNAc)_3_. Reactions were carried out in 96-well plates for 30 min at 37°C and were terminated by adding a stop solution (400 mM sodium carbonate). Fluorescence was measured at an excitation wavelength of 360 nm and an emission wavelength of 450 nm, in an Infinite Series M200 microplate spectrophotometer (Tecan Trading AG, Switzerland), no later than 30 min after ending the reaction. One unit of chitinase activity was defined as the release of 1 µmol of 4-MUF per minute at assay conditions. Effects of pH on activity were determined from pH 3–11 at standard conditions using 25 mM of each buffer, sodium citrate (pH 3), sodium acetate (pH 4–5), sodium phosphate (pH 6–7), Tris-HCl (pH 8–9) and sodium carbonate (pH 10–11). The effects of temperature on activity were determined between 20° and 70°C for 10 to 30 min at pH 5.

### Chitinase activity assay using colloidal chitin

Reactions consisted of 400 µL of 10 g/L colloidal chitin in sodium acetate buffer, pH 5, and 40 µg of enzyme. Standard conditions of incubation were pH 5 and 37°C for 30 min. After incubation, the samples were centrifuged at 12,000 *g* for 5 min, 100 µL of supernatant was added to 100 µL of DNS reagent (25), boiled for 20 min, cooled at room temperature and read at 550 nm. The standard curve was determined with N-acetylglucosamine from 50 to 800 nM. One unit of chitinase activity was defined as the release of 1 µmol of reducing sugar per minute at assay conditions. The pH range used in activity and stability tests was from 3 to 11 and the temperature range was from 20° to 70°C. Standard conditions were used for activity tests, with varying pH or temperature values in the respective assays. After enzyme incubation for 1, 3 or 24 h at varying pH or temperature values, the remaining activity of MetaChi18A was detected using standard conditions. All buffers used were at a concentration of 25 mM: sodium carbonate (pH 11 and 10), Tris-HCl (pH 9 and 8), sodium phosphate (pH 7 and 6), sodium acetate (pH 5 and 4), and sodium citrate (pH 3). Metal ions were added at 1, 5, or 10 mM final concentrations and chloride salts of Al^3+^, Ca^2+^, Co^2+^, Cu^2+^, Fe^3+^, Li^+^, Mg^2+^, Mn^2+^, Sn^2+^, and Zn^2+^ were used.

### Substrate specificity

MetaChi18A activity was determined towards chitosans with a degree of acetylation (DA) of 10% (MW∼82 kDa), 20% (MW∼85 kDa), 35% (MW∼88 kDa), 50% (MW∼91 kDa), and 60% (MW∼93 kDa); all chitosan polymers had an average degree of polymerization (DP) of 500. And also towards colloidal chitin (DP∼1000, MW∼161 kDa), α- and β-chitin, activity was determined following the method of Horn and Eijsink (26). Reactions consisted of 40 µL of 1 g/L substrate in sodium acetate buffer (pH 5), 1 ng/µL of enzyme and incubation at pH 5 and 50°C for 1.5 h.

### High-performance thin layer chromatography (HP-TLC)

HP-TLC was used to analyze the oligomers resulting from the enzymatic hydrolysis of commercial dimers to hexamers of GlcNAc A2-A6 (Megazyme, Ireland). Reactions were performed with 1.25 mg/mL of each oligomer as a substrate (in 10 mM ammonium acetate buffer pH 5) and 0.75 mg/mL of MetaChi18A, for 5 to 60 min at 50°C. Mixtures containing the hydrolyzed products were loaded onto silica gel coated HP-TLC plates (Merck, Germany), carefully dried, run against the solvent (n-butanol, methanol, 25% ammonia, and water in a ratio of 5:4:2:1) in a chromatography chamber until the solvent front reached 3/4 of the TLC plate. The plate was dried and dipped into the staining solution (30% ammonium bisulphate in water) followed by heating at 180°C (hot air gun, Black and Decker, Germany) to develop the spots on the TLC plate. Commercial glucosamine and N-acetylglucosamine oligomers (Carbosynth, UK and Megazyme, Ireland) were used as standards.

### Product analysis by mass spectrometry

Standard reactions were incubated for 15 min, 30 min, 1, 5, and 24 h. Matrix-assisted laser desorption/ionization (MALDI) MS analysis was carried out using the protocol of Price and Naumann (27). Equal volumes of matrix [saturated 2,5-dihydroxybenzoic acid (2,5-DHB) in acetonitrile] and reaction supernatant were spotted onto the MALDI plaque and left to dry. Mass spectra were recorded on a Bruker Daltonic OmniF instrument (USA) operating in reflection mode of positive ions, with an acceleration voltage of 20 kV, 150 ns. The MS spectra were recorded in triplicates with matrix suppression from m/z 200 to m/z 800. Excitation was set at circa 50% of maximum output and 250 shots were accumulated.

### Kinetic parameters determination

Steady-state kinetic data for MetaChi18A were obtained using as substrate colloidal chitin from 0.5 to 10 mg/mL or 4-MUF-(GlcNAc)_2_ from 1.25 to 20 µM and 1 µM of enzyme. Kinetic parameters were determined by measuring the initial rates of reaction from the increase of reducing sugar concentration, as determined using the DNS reaction, using colloidal chitin as substrate, and from the increase of MUF concentration using 4-MUF-(GlcNAc)_2_ as substrate. The values of the kinetic constants were calculated by non-linear least-square regression, fitting the data to equation 1, for a multiple substrate enzyme complex (28).



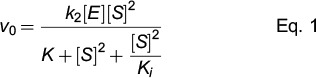



where v_0_ is the reaction rate, k_2_ a rate constant, [E] enzyme concentration, [S] substrate concentration, and K and K_i_ are dissociation constants. GraphPadPrism 5 software was used to calculate regressions, including allosteric kinetics (V_0_=Vmax*[S]^h^/(K+ [S]^h^), where h is the Hill slope, Michaelis-Menten (V_0_ = Vmax*[S]/(Km + [S]) and MM with substrate inhibition (V_0_=Vmax*[S]/(Km + [S]*(1+[S]/Ki).

### Statistical analysis

All experiments were performed with technical and biological triplicates. Data are reported as means and standard deviations.

### Nucleotide sequence accession number

The nucleotide sequence of *metachi18A* was deposited at the GenBank database under accession No. KJ160494.

## Results

### Activity screening and sequence analysis

**Figure 1:**
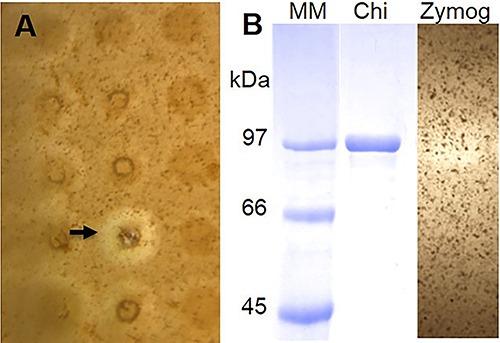
Screening and purification of chitinase MetaChi18A. *A*, Hydrolysis halo produced by MetaChi18A metagenomic clone (arrow) on colloidal chitin agar plate. *B*, SDS-PAGE of purified MetaChi18A. MM: molecular weight marker (kDa); Chi: purified MetaChi18A stained with Coomassie blue; Zymog: zymogram showing chitinolytic activity of purified MetaChi18A on colloidal chitin.

A metagenomic library (approximately 500,000 clones), hosted in *E. coli* and constructed with DNA isolated from a wastewater treatment plant (15), was screened for chitinase activity. A pool of the *E. coli* cells harboring the whole metagenomic library was plated on LB Agar containing 2% colloidal chitin (Figure 1A). Fifteen of 50,000 screened colonies presented chitinolytic activity, their fosmids were purified, and the restriction cut analyses showed one single pattern. After re-transformation of *E. coli* EPI300 with the single fosmid, the clone kept the chitinolytic activity.

The fosmid clone conferring chitinase activity was purified, mechanically fragmented by nebulization, and fragments of 3 and 5 kb were separated and subcloned into the vector pUC18. A chitin-hydrolyzing subclone with a 3 kb insert was sequenced revealing an open reading frame (ORF) of 2594 nucleotides coding for a protein with a theoretical pI of 5.12 and molecular weight of 92 kDa. This ORF, named *metachi18A*, shares 99% amino acid identity with a glycosyl hydrolase from *Aeromonas jandaei* [RefSeq: WP_042029877.1] not yet characterized biochemically. The closest characterized enzyme is a chitinase from *Aeromonas hydrophila* [DBJ: BAE87051.1] with 83% amino acid identity (29).

**Figure 2:**
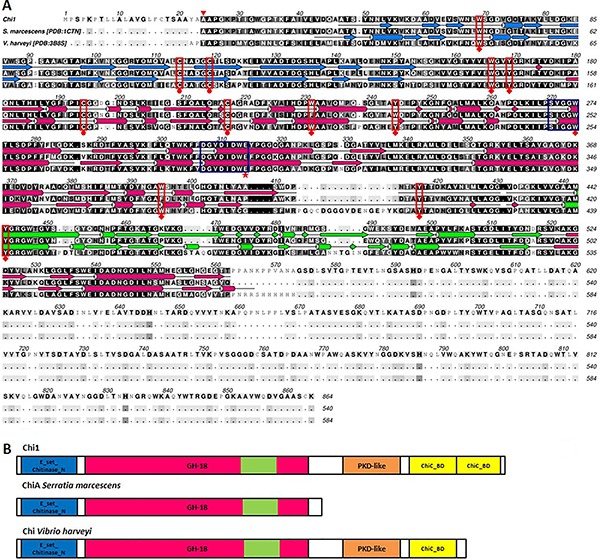
Amino acid sequence alignment, secondary structure estimation and domains schematic representation of MetaChi18A and most similar available chitinases structures. *A*, The triangle (pointing down) indicates the cleavage site of the signal peptide, diamonds indicate the conserved aromatic residues binding the substrate, circles indicate disulfide bonds and the star indicates the glutamate residue involved in the hydrolysis. Dark blue boxes indicate the conserved sequences in the catalytic domain from glycosyl hydrolases family 18. The sequence background is colored according to the convention ALSCRIPT Calcons. Representations of secondary structures are given above the sequences: arrows for β-strands and cylinders for α-helix. Light blue is the E_set_chitinase_N, N-terminal domain, pink is the catalytic domain, and green is the (α+β) insertion in catalytic domain. *B*, Domain representations follow the same colors with the addition of: orange - PKD (polycystic kidney disease)-like domain with Ig-like fold, which probably functions as a ligand-binding site in protein-protein or protein-carbohydrate interactions and yellow - ChiC_BD (chitin-binding domain related to ChiC of *Streptomyces griseus*). The N-terminal domain and the GH-18 catalytic domain from MetaChi18A [Genbank: KJ160494] have 74% identity with ChiA from *Serratia marcescens* [GenBank: ABI79317.1] and 54% identity with the chitinase from *Vibrio harveyi* [GenBank: AIV07901.1].

Phylogenetic analysis (Neighbor-Joining method) placed MetaChi18A in the *Aeromonas* chitinases group of glycosyl hydrolases family 18 (GH-18) (data not shown). *Serratia marcescens* ChiA is the closest, structurally related, characterized enzyme (74% identity on amino acid level in the N-terminal and catalytic domain) (GenBank: ABI79317.1; Figure 2). The metagenomic MetaChi18A possesses an N-terminal chitin binding domain E_set_chitinase_N, that is a fibronectin type 3-like domain (Fn3), followed by the GH-18 domain and two C-terminal chitin-binding domains (ChiC_BD) joined to the GH-18 domain by a Polycystic Kidney Disease-like domain (PKD) (Figure 2). The N-terminus contains a 23 amino acid signal peptide with a predicted cleavage site between residues Ala23 and Ala24. The modeling of MetaChi18A on the basis of the known crystal structure of *S. marcescens* ChiA (data not shown) predicts that it has a deep tunnel-like active site groove with an additional (α+β) domain typical for subfamily A of GH-18 catalytic domains.

### Overexpression and purification of the recombinant MetaChi18A chitinase

The *metachi18A* gene was PCR-amplified without the signal peptide and cloned into the pET28a expression vector. The MetaChi18A protein carrying a C-terminal 6xHis-tag was expressed in *E. coli* BL21 (DE3) and purified by affinity chromatography. Most of the expressed protein was insoluble (about 70% of total expressed MetaChi18A) but the protein in soluble fraction yielded 180 µg of pure MetaChi18A per liter of *E. coli* culture. We chose to repeat the purification to get more chitinase rather than adding components to solubilize more protein, because they could interfere on further experiments for MetaChi18A characterization. The MetaChi18A preparation was homogeneous as analyzed by SDS-PAGE (Figure 1B). Zymographic analysis using colloidal chitin as substrate showed a clear band around the 97 kDa region revealing that the purified enzyme was active (Figure 1B) and the molecular mass was as predicted from the translated nucleotide sequence. Peptide mass fingerprinting by MALDI mass spectrometry confirmed that the purified enzyme was indeed MetaChi18A (data not shown).

### Substrate specificity

Substrates 4-methylumbelliferyl N-acetyl-β-D-glucosamine (MUF-GlcNAc), 4-methylumbelliferyl N,N′-diacetylchitobiose (4-MUF-(GlcNAc)_2_) and 4-methylumbelliferyl β-D-N,N′,N′′-triacetylchitotriose (4-MUF-(GlcNAc)_3_) were used for a preliminary characterization of MetaChi18A specificity. MetaChi18A did not show detectable activity towards 4-MUF-GlcNAc, but the activity was 0.031 and 0.015 U/mg for 4-MUF-(GlcNAc)_2_ and 4-MUF-(GlcNAc)_3_ at 37°C, respectively. MetaChi18A was also active against colloidal chitin with an activity of 0.018 U/mg at 50°C and 0.008 U/mg at 37°C.

**Figure 3:**
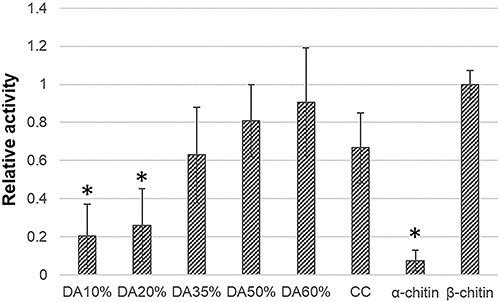
Substrate specificity of MetaChi18A. Activities relative to the highest activity (135 U/mg on β-chitin) measured through quantification of reducing ends in a reaction with 1 g/L substrate in sodium acetate buffer, pH 5, 1 ng/µL of enzyme and incubation at pH 5 and 50°C for 1.5 h. DA: degree of acetylation of chitosan; CC: colloidal chitin. Data are reported as the average of 3 independent experiments with standard errors. *P<0.05, compared to β-chitin activity (*t*-test).

MetaChi18A was capable of hydrolyzing chitins and chitosans of different degrees of solubility and acetylation (Figure 3). MetaChi18A had higher activity towards more acetylated chitosan and β-chitin, but it was not able to hydrolyze α-chitin at tested incubation times.

### Effect of pH and temperature on MetaChi18A activity

**Figure 4:**
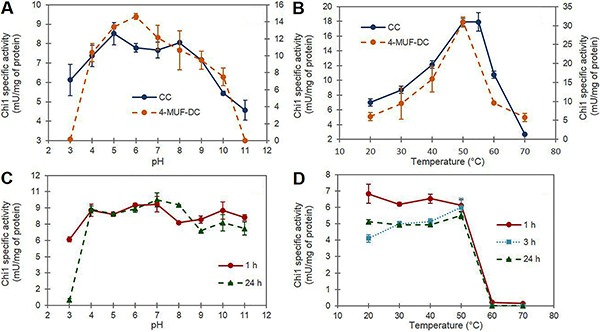
Effect of pH and temperature on MetaChi18A activity and stability. Chitinolytic activity was determined using 0.2 mg/mL of 4-MUF derivatives (*A* and *B* orange slashed line, Y-axis on the right) and 10 mg/mL of colloidal chitin (*A* and *B* dark blue line, Y-axis on the left) as substrate. Enzyme concentration was 0.40 and 100 ng/µL, respectively. *A*, Effect of pH on the activity for 30 min at 37°C; *B*, effect of temperature on the activity for 30 min at pH 5; *C*, stability of MetaChi18A incubated at indicated pH for 1 or 24 h without substrate; *D*, stability of MetaChi18A incubated at the indicated temperature for 1, 3, or 24 h without substrate. The remaining activities were measured for 30 min at 37°C and pH 5 using 10 mg/mL of colloidal chitin. The reactions were performed in 25 mM buffers: pH 3 sodium citrate; pH 4 and 5 sodium acetate; pH 6 and 7 sodium phosphate; pH 8 and 9 Tris-HCl; pH 10 and 11 sodium carbonate. Error bars represent standard deviation. One unit of chitinase activity (U) was defined as the release of 1 µmol of reducing sugar or 4-MUF per minute.

MetaChi18A has a very broad pH optimum on both 4-MUF-(GlcNAc)_2_ and colloidal chitin used as substrate, ranging from 4 to 9 (Figure 4A and C). The activity of MetaChi18A on colloidal chitin was higher than 80% of the maximum activity in the wide pH range from 4 to 9, with a peak at pH 5. Moreover, incubation in buffers, with pH from 4 to 11 for up to 24 h at room temperature did not inactivate the enzyme.

The optimum activity for MetaChi18A in a 30 min reaction was at 50°C using both 4-MUF-(GlcNAc)_2_ and colloidal chitin. Over 40% of the enzyme was inactivated at 60°C and had low activity below 40°C (Figure 4B). Incubation at 50°C for 3 h did not cause any decrease in activity. Even after 24 h of incubation at this temperature, MetaChi18A kept 60% of its activity. However, incubation at 60°C or higher temperatures led to rapid inactivation (Figure 4D).

### Activity assay in the presence of metal ions, EDTA, SDS, Triton X-100 and NaCl

The effect of metal ions on MetaChi18A activity was determined at a concentration of 1 mM (Table 1). Sn^2+^ and Mg^2+^ slightly decreased MetaChi18A’s activity. At higher concentrations (5 and 10 mM) only Al^3+^ substantially reduced ChiA activity by 50 and 95%, respectively. MetaChi18A retained 98% of its activity in 0.6 M NaCl and 77% in 30 mM EDTA. The addition of detergents such as Triton X-100 up to 5% did not affect MetaChi18A activity, while 0.1% SDS and a urea concentration of over 1 M decreased the activity by 25% (Table 1). All activities in Table 1 are relative to the control reaction, performed under standard conditions without any additive.

**Table:**
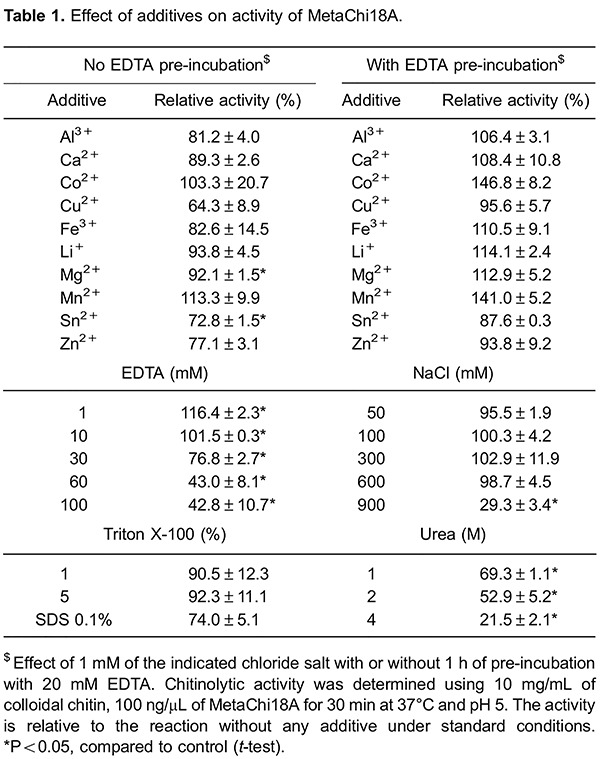

### Identification of chitin hydrolysis product

**Figure 5:**
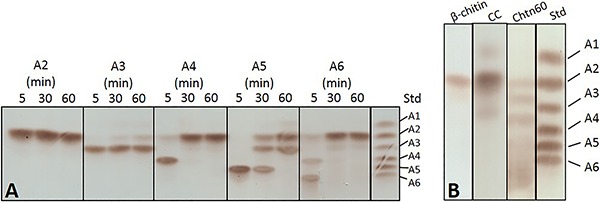
Products of MetaChi18A activity on different substrates: chitooligomers (A2-A6) and polymers; β-chitin, colloidal chitin (CC) and chitosan 60% acetylated (Chtn60). MetaChi18A hydrolysis products were separated by thin-layer chromatography. *A*, Reaction with 0.76 ng/µL MetaChi18A, 1.25 g/L oligomers in 100 mM MES buffer, pH 5, at 50°C for 5, 30, and 60 min. *B*, reaction with 1 ng/µL MetaChi18A, 1 g/L β-chitin, CC or Chtn60 in 10 mM ammonium acetate buffer, pH 5, at 50°C for 24 h.

A time course TLC analysis of MetaChi18A reaction with different substrates indicated that MetaChi18A does not hydrolyze GlcNAc dimers (A2) (Figure 5A). Trimers (A3) are poorly cleaved into dimers and the monomer GlcNAc. MetaChi18A hydrolyzed A5 initially to produce A3 and A2. The even numbered substrates, A4 and A6, were cleaved into A2, with A6 being first cleaved into A4 and a small amount into A3 (Figure 5A).

MetaChi18A was not able to cleave deacetylated oligomers (D2-D6; data not shown). Chitin related polymers with decreasing degree of solubility such as chitosan, colloidal chitin and β-chitin (Figure 5B) produced a series of small oligomers. However, the latter two substrates produced mainly dimers. MALDI-TOF-MS analyses identified only diacetylchitobiose as the product of colloidal chitin cleaved by MetaChi18A (data not shown).

### Kinetic parameters

**Figure 6:**
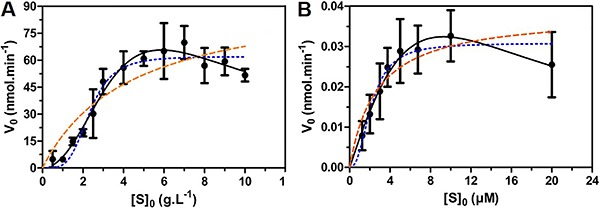
Kinetic behavior of MetaChi18A. *A*, Colloidal chitin as substrate; *B*, 4-MUF-(GlcNAc)_2_ as substrate. Colloidal chitin (g/L) and V_0_ (nmol of reducing sugar/min); 4-MUF-(GlcNAc)_2_ (µM) and V_0_ (nmol of MUF/min). Reactions were performed at 25 mM sodium acetate buffer, pH 5, at 50°C (*A*) or 37°C (*B*). The black line is the model that better fits the data: (*A*) allosteric kinetic+substrate inhibition, and (*B*) substrate inhibition; Blue dotted line: allosteric kinetic; Orange slashed line: Michaelis-Menten kinetic.

The kinetic constants of MetaChi18A were determined with colloidal chitin as substrate at 50°C, or 4-MUF-(GlcNAc)_2_ at 37°C. High concentrations of both substrates inhibited MetaChi18A activity (Figure 6). In addition, MetaChi18A exhibited a sigmoidal kinetic with colloidal chitin (Figure 6A).

Different kinetic models accounting for substrate inhibition or for allosteric behavior were used to determine the kinetic parameters (Table 2) of MetaChi18A. The model used for ChiA from *Serratia marcescens* (Equation 1) fit satisfactorily to the experimental data with colloidal chitin, while for 4-MUF kinetics the Michaelis-Menten equation for substrate inhibition fit better (Figure 6B). The K_M_, v_max_ and K_I_ or Hill slope values were calculated using non-linear regression analysis available on GraphPad Prism 5 or using Equation 1, and the values for both substrates are reported in Table 2.

**Table:**
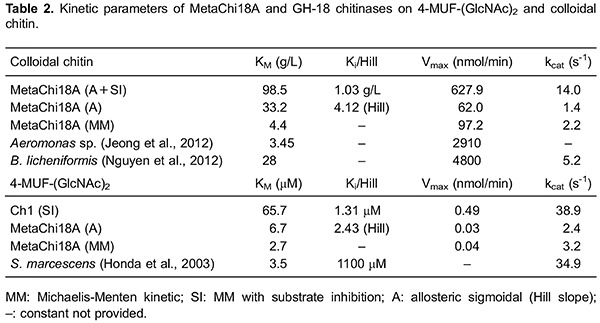

## Discussion

Functional screening of metagenomic libraries for chitinases is still a challenge due to the heterologous expression and to slow substrate degradation. Until now, metagenomic libraries were screened for chitinases using sequence-based methods based on PCR or NGS, or functional screening methods using artificial chitin oligomer derivatives, that are more easily degraded. In our work, we were able to surmount these difficulties by screening a metagenomic fosmid library that was produced by indirect DNA extraction, therefore with high prokaryotic DNA content, and incubating plates of the substrate (colloidal chitin) and fosmid clones for a longer time (almost 2 months.) Even though we found 15 active clones, the search resulted in a single fosmid. We managed to save time and material by screening the pool of cells containing the whole metagenomic library. Although an estimate of 50,000 clones were checked, the redundancy of chitinase active clones shows that further screening would probably not lead to a new clone discovery.

Metcalfe et al. (30) showed that the diversity of the chitinolytic community of an upland pasture soil decreased after treatment with domestic sludge, although it increased the chitinolytic activity. The readily available carbon and nitrogen sources did not repress chitinases but stimulated the activity of specific groups of chitinolytic actinobacteria. As our metagenomic source was the soil of an industrial waste treatment lagoon, the same effect could result in the invariability of the chitinolytic clones we found.

In spite of the high similarity with other Aeromonas chitinases, MetaChi18A showed distinguished features when compared with the closest characterized chitinase from *Aeromonas hydrophila* (29). The latter has a pH optimum between 5 and 7.5 and shows higher activity at 42°C. Its relative activity on chitosan DA 20% is only 7% lower than on colloidal chitin, while MetaChi18A has low activity on chitosan DA 20%. Its diverse subsite specificities are also evident in the products of oligomers hydrolysis. *A. hydrophila* chitinase completely hydrolyzes the trimer into dimer and monomer, while MetaChi18A poorly hydrolyzes it. This suggests that a few amino acid modifications can lead to significantly divergent hydrolysis features.

The structural model together with sequence analysis support the conclusion that MetaChi18A acts processively on its substrate generating diacetylchitobiose as the main product, as reported for ChiA and ChiB from *S. marcescens* (31). Indeed, TLC and MALDI-TOF analyses identified chitobiose as the single MetaChi18A product of β-chitin and the main product of colloidal chitin hydrolysis. Moreover, MetaChi18A presents typical features of processive enzymes; the insertion domain (α+β) at the catalytic domain, a stretch of aromatic amino acid residues on its surface, from the N-terminal substrate-binding domain to the catalytic domain, and the conserved residues Trp166 (corresponding to Trp167 in ChiA from *S. marcescens*), Trp274 (corresponding to Trp275 in ChiA) and Trp395 (corresponding to Phe396 in ChiA) (32).

Most chitinases described to date have high activity at acidic or close to neutral pH. MetaChi18A has more than 80% of activity from pH 4 to 9 and remains active at 50°C for 24 h. The results suggest that MetaChi18A is moderately thermophilic, as it would be expected since it is similar to *Aeromonas* chitinases. *Aeromonas* sp. can be found inhabiting natural soil, food and animals, but mostly all kinds of aquatic environments. The mesophilic species have optimal growth at 35° to 37°C (33).

Bacterial chitinases respond differently to the presence of a variety of metal ions and MetaChi18A was not highly affected by the metal ions tested. It has been reported that the inhibition of chitinase by certain divalent cations occurs because they are able to form stable complexes with carboxylic groups of aspartic and glutamic acid residues at the active site (34), but activity assays with EDTA and all tested ions showed that MetaChi18A has no necessity of a cofactor for its activity.

Concerning the resilience to higher concentrations of NaCl, MetaChi18A remained active until 600 mM. We cannot claim that this characteristic is related only to the presence of high NaCl concentrations in the original environment, as a wastewater treatment lagoon is a rich medium with high concentration of nutrients and microorganisms. MetaChi18A showed high stability on different conditions including high activity in a larger pH range. This may be due to the additional domains at both N- and C-terminals that keep its structure more stable avoiding its disturbance in these conditions. Such higher stability was already described for chitinases containing chitin binding domain and Fn3 domain (35,36) but most chitinases have one or two of these domains in one side while our MetaChi18A has four additional domains surrounding the catalytic domain, which might protect even more its integrity.

As usual for chitinases of the GH-18 family, MetaChi18A had higher activity towards more acetylated substrates. Since the enzyme requires an acetylated glucosamine residue positioned in the -1 catalytic subsite (5), the higher the degree of acetylation (DA) the higher the abundance of cleavage sites in the polymeric substrates. MetaChi18A was not able to hydrolyze α-chitin presumably due to its crystalline structure with highly packed chains making access to the enzyme difficult. It was described that the lytic polysaccharide monooxygenases facilitate access into α-chitin chains by producing oxidative cuts along the chain (3).

Although Cruys-Bagger et al. (37) described that processive enzymes such as cellulases also show a hyperbolic relationship between steady-state rate and substrate concentration, the kinetic parameters are more complex and may not be calculated directly from the Michaelis-Menten equation. Kinetics of ChiA from *S. marcescens* on 4-MUF-(GlcNAc)_2_ had the same profile and its parameters were determined using Equation 1 (28). MetaChi18A and ChiA from *S. marcescens* showed similar catalytic constants (k_cat_): 38.9 and 34.9 s^-1^, respectively. On colloidal chitin, the kinetic parameters of *Aeromonas* sp. GJ 18 and *Bacillus licheniformis* chitinases were determined using Michaelis-Menten non-linear regression (38,39). The K_M_ for MetaChi18A was similar to that of *Aeromonas* chitinase while the k_cat_ value was similar to that of *Bacillus* chitinase (Table 2).

Many chitinases of family GH-18 exhibit substrate inhibition by substrates of low molecular weight such as 4-MUF-(GlcNAc)_2_. The proposed MetaChi18A active cleft contains multi-subsites where small substrates could bind differently leading to non-productive inhibitory binding (40). However, this inhibitory mechanism would not be expected for inhibition caused by high concentrations of the polymeric colloidal chitin, where inhibition is more probably caused by impaired diffusion of the enzyme. MetaChi18A showed a Hill slope of 4, indicating substrate-binding cooperativity. This phenomenon may occur due to hysteresis, a delay of the enzyme in reaching its fully active form that involves conformational changes. As MetaChi18A is a multi-domain enzyme, the substrate should interact with binding domains that could rearrange the conformation to facilitate access of the catalytic domain to the substrate.

Although most kinetic studies on chitinases have used small oligomers as substrates, not reflecting their native behavior on chitin, our study is one of few (38,39) that report kinetics on colloidal chitin. The data obtained reflect better the natural substrate, and help to elucidate how chitinases behave on polymeric substrates.

Our study was successful in the identification of a chitinolytic clone by functional screening of a metagenomic fosmid library. We have succeeded in the MetaChi18A biochemical and kinetic characterization, using colloidal chitin, which is a more soluble formulation of polymeric chitin.

Current limitations on the use of chitinases for biotechnological applications are their low stability and the limited range of temperatures and pH values in which the chitinases described to date are functional. The MetaChi18A chitinase described in this study is a promising alternative for industrial processing of chitin as judged by its high stability and activity under a broad range of pH values and temperatures.

## References

[1] Gooday GW (1990). Physiology of microbial degradation of chitin and
chitosan. Biodegradation.

[2] Yan N, Chen X (2015). Sustainability: Don't waste seafood waste. Nature.

[3] Vaaje-Kolstad G, Westereng B, Horn SJ, Liu Z, Zhai H, Sorlie M (2010). An oxidative enzyme boosting the enzymatic conversion of recalcitrant
polysaccharides. Science.

[4] Henrissat B, Bairoch A (1993). New families in the classification of glycosyl hydrolases based on
amino acid sequence similarities. Biochem J.

[5] van Aalten DM, Komander D, Synstad B, Gaseidnes S, Peter MG, Eijsink VG (2001). Structural insights into the catalytic mechanism of a family 18
exo-chitinase. Proc Natl Acad Sci U S A.

[6] Li H, Greene LH (2010). Sequence and structural analysis of the chitinase insertion domain
reveals two conserved motifs involved in chitin-binding. PLoS One.

[7] Sørlie M, Zakariassen H, Norberg AL, Eijsink VGH (2012). Processivity and substrate-binding in family 18
chitinases. Biocatal Biotransformation.

[8] Leresche JE, Meyer H-P (2006). Chemocatalysis and biocatalysis (biotransformation): some thoughts of
a chemist and of a biotechnologist. Org Process Res Dev.

[9] Torsvik V, Ovreas L (2002). Microbial diversity and function in soil: from genes to
ecosystems. Curr Opin Microbiol.

[10] Handelsman J, Rondon MR, Brady SF, Clardy J, Goodman RM (1998). Molecular biological access to the chemistry of unknown soil microbes:
a new frontier for natural products. Chem Biol.

[11] Stoveken J, Singh R, Kolkenbrock S, Zakrzewski M, Wibberg D, Eikmeyer FG (2015). Successful heterologous expression of a novel chitinase identified by
sequence analyses of the metagenome from a chitin-enriched soil
sample. J Biotechnol.

[12] Beier S, Jones CM, Mohit V, Hallin S, Bertilsson S (2011). Global phylogeography of chitinase genes in aquatic
metagenomes. Appl Environ Microbiol.

[13] Cretoiu MS, Kielak AM, Abu Al-Soud W, Sorensen SJ, van Elsas JD (2012). Mining of unexplored habitats for novel chitinases - chiA as a helper
gene proxy in metagenomics. Appl Microbiol Biotechnol.

[14] Hsu SC, Lockwood JL (1975). Powdered chitin agar as a selective medium for enumeration of
actinomycetes in water and soil. Appl Microbiol.

[15] Glogauer A, Martini VP, Faoro H, Couto GH, Muller-Santos M, Monteiro RA (2011). Identification and characterization of a new true lipase isolated
through metagenomic approach. Microb Cell Fact.

[16] Ewing B, Hillier L, Wendl MC, Green P (1998). Base-calling of automated sequencer traces using phred. I. Accuracy
assessment. Genome Res.

[17] Bendtsen JD, Nielsen H, von Heijne G, Brunak S (2004). Improved prediction of signal peptides: SignalP 3.0. J Mol Biol.

[18] Gasteiger E, Gattiker A, Hoogland C, Ivanyi I, Appel RD, Bairoch A (2003). ExPASy: The proteomics server for in-depth protein knowledge and
analysis. Nucleic Acids Res.

[19] Thompson JD, Higgins DG, Gibson TJ (1994). CLUSTAL W: improving the sensitivity of progressive multiple sequence
alignment through sequence weighting, position-specific gap penalties and weight
matrix choice. Nucleic Acids Res.

[20] Berman HM, Kleywegt GJ, Nakamura H, Markley JL (2014). The Protein Data Bank archive as an open data resource. J Comput Aided Mol Des.

[21] Kabsch W, Sander C (1983). Dictionary of protein secondary structure: pattern recognition of
hydrogen-bonded and geometrical features. Biopolymers.

[22] Bond CS, Schuttelkopf AW (2009). ALINE: a WYSIWYG protein-sequence alignment editor for
publication-quality alignments. Acta Crystallogr D Biol Crystallogr.

[23] Tronsmo A, Harman GE (1993). Detection and quantification of N-acetyl-beta-D-glucosaminidase,
chitobiosidase, and endochitinase in solutions and on gels. Anal Biochem.

[24] Westermeier R, Loyland S, Asbury R (2002). Proteomics technology. J Clin Ligando Assay.

[25] Miller GL (1959). Use of dinitrosalicylic acid reagent for determination of reducing
sugar. Anal Chem.

[26] Horn SJ, Eijsink VGH (2004). A reliable reducing end assay for
chito-oligosaccharides. Carbohydr Polym.

[27] Price NP, Naumann TA (2011). A high-throughput matrix-assisted laser
desorption/ionization-time-of-flight mass spectrometry-based assay of chitinase
activity. Anal Biochem.

[28] Honda Y, Kitaoka M, Tokuyasu K, Sasaki C, Fukamizo T, Hayashi K (2003). Kinetic studies on the hydrolysis of N-acetylated and N-deacetylated
derivatives of 4-methylumbelliferyl chitobioside by the family 18 chitinases ChiA
and ChiB from *Serratia marcescens*. J Biochem.

[29] Lan X, Zhang X, Hu J, Shimosaka M (2006). Cloning, expression, and characterization of a chitinase from the
chitinolytic bacterium *Aeromonas hydrophila* strain
SUWA-9. Biosci Biotechnol Biochem.

[30] Metcalfe AC, Krsek M, Gooday GW, Prosser JI, Wellington EM (2002). Molecular analysis of a bacterial chitinolytic community in an upland
pasture. Appl Environ Microbiol.

[31] Sikorski P, Sorbotten A, Horn SJ, Eijsink VG, Varum KM (2006). *Serratia marcescens* chitinases with tunnel-shaped
substrate-binding grooves show endo activity and different degrees of processivity
during enzymatic hydrolysis of chitosan. Biochemistry.

[32] Payne CM, Baban J, Horn SJ, Backe PH, Arvai AS, Dalhus B (2012). Hallmarks of processivity in glycoside hydrolases from
crystallographic and computational studies of the Serratia marcescens
chitinases. J Biol Chem.

[33] Janda JM, Abbott SL (2010). The genus *Aeromonas*: taxonomy, pathogenicity, and
infection. Clin Microbiol Rev.

[34] Milewski S, O'Donnell RW, Gooday GW (1992). Chemical modification studies of the active centre of *Candida
albicans* chitinase and its inhibition by allosamidin. J Gen Microbiol.

[35] Lin FP, Juang WY, Chang KH, Chen HC (2001). G561 site-directed deletion mutant chitinase from *Aeromonas
caviae* is active without its 304 C-terminal amino acid
residues. Arch Microbiol.

[36] Sha L, Shao E, Guan X, Huang Z (2016). Purification and partial characterization of intact and truncated
chitinase from *Bacillus thuringiensis* HZP7 expressed in
*Escherichia coli*. Biotechnol Lett.

[37] Cruys-Bagger N, Elmerdahl J, Praestgaard E, Borch K, Westh P (2013). A steady-state theory for processive cellulases. FEBS J.

[38] Nguyen HA, Nguyen TH, Nguyen TT, Peterbauer CK, Mathiesen G, Haltrich D (2012). Chitinase from *Bacillus licheniformis* DSM13:
expression in *Lactobacillus plantarum* WCFS1 and biochemical
characterisation. Protein Expr Purif.

[39] Jeong HC, Ju W-T, Jo K-H, Park RD (2012). Purification and characterization of a 34-kDa chitobiosidase from
*Aeromonas* sp. GJ-18. J Korean Soc Appl Biol Chem.

[40] Honda Y, Kirihata M, Fukamizo T, Kaneko S, Tokuyasu K, Brzezinski R (1999). Chitosanase-catalyzed hydrolysis of 4-methylumbelliferyl
beta-chitotrioside. J Biochem.

